# MARCKS Signaling Differentially Regulates Vascular Smooth Muscle and Endothelial Cell Proliferation through a KIS-, p27^kip1^- Dependent Mechanism

**DOI:** 10.1371/journal.pone.0141397

**Published:** 2015-11-03

**Authors:** Dan Yu, George Makkar, Tuo Dong, Dudley K. Strickland, Rajabrata Sarkar, Thomas Stacey Monahan

**Affiliations:** 1 Department of Surgery, Baltimore Veterans Affairs Medical Center, Baltimore, Maryland, United States of America; 2 Department of Surgery, University of Maryland School of Medicine, Baltimore, Maryland, United States of America; 3 Department of Physiology, University of Maryland School of Medicine, Baltimore, Maryland, United States of America; 4 Center for Vascular and Inflammatory Diseases, University of Maryland School of Medicine, Baltimore, Maryland, United States of America; Nagoya University, JAPAN

## Abstract

**Background:**

Overexpression of the myristolated alanine-rich C kinase substrate (MARCKS) occurs in vascular proliferative diseases such as restenosis after bypass surgery. MARCKS knockdown results in arrest of vascular smooth muscle cell (VSMC) proliferation with little effect on endothelial cell (EC) proliferation. We sought to identify the mechanism of differential regulation by MARCKS of VSMC and EC proliferation *in vitro* and *in vivo*.

**Methods and Results:**

siRNA-mediated MARCKS knockdown in VSMCs inhibited proliferation and prevented progression from phase G_0_/G_1_ to S. Protein expression of the cyclin-dependent kinase inhibitor p27^kip1^, but not p21^cip1^ was increased by MARCKS knockdown. MARCKS knockdown did not affect proliferation in VSMCs derived from p27^kip1^-/- mice indicating that the effect of MARCKS is p27^kip1^-dependent. MARCKS knockdown resulted in decreased phosphorylation of p27^kip1^ at threonine 187 and serine 10 as well as, kinase interacting with stathmin (KIS), cyclin D1, and Skp2 expression. Phosphorylation of p27^kip1^ at serine 10 by KIS is required for nuclear export and degradation of p27^kip1^. MARCKS knockdown caused nuclear trapping of p27^kip1^. Both p27^kip1^ nuclear trapping and cell cycle arrest were released by overexpression of KIS, but not catalytically inactive KIS. In ECs, MARCKS knockdown paradoxically increased KIS expression and cell proliferation. MARCKS knockdown in a murine aortic injury model resulted in decreased VSMC proliferation determined by bromodeoxyuridine (BrdU) integration assay, and inhibition of vascular wall thickening. MARCKS knockdown increased the rate of re-endothelialization.

**Conclusions:**

MARCKS knockdown arrested VSMC cell cycle by decreasing KIS expression. Decreased KIS expression resulted in nuclear trapping of p27^kip1^ in VSMCs. MARCKS knockdown paradoxically increased KIS expression in ECs resulting in increased EC proliferation. MARCKS knockdown significantly attenuated the VSMC proliferative response to vascular injury, but accelerated reestablishment of an intact endothelium. MARCKS is a novel translational target with beneficial cell type-specific effects on both ECs and VSMCs.

## Introduction

Excessive vascular smooth muscle cell (VSMC) proliferation is the hallmark of pathologic vascular proliferative disorders such as restenosis after angioplasty and stenting or bypass surgery. The majority of the cellular mass in restenotic vascular lesions is comprised of dedifferentiated VSMCs originating from the media. The iatrogenic trauma causes these cells to revert from their quiescent, contractile phenotype to a migratory, secretory, and proliferative phenotype [1,2]. Drug-eluting stents and drug-coated balloons are used to prevent VSMC proliferation. Unfortunately, these anti-proliferative agents inhibit both VSMC and endothelial cell (EC) proliferation. Drug-eluting stents actually have an increased risk of late thrombosis compared to bare metal stents, which is likely due to the failure of the treated artery to re-endothelialize [3,4]. As a result, patients with drug-eluting stents have to remain on dual antiplatelet therapy indefinitely with associated increased bleeding complications. An ideal strategy to prevent restenosis in cardiovascular surgery would selectively inhibit VSMC proliferation without interfering with EC proliferation and re-endothelialization.

The myristolated alanine-rich C kinase substrate (MARCKS) is upregulated at early time points in large animal models of vein graft [5] and prosthetic graft [6] bypass surgery. These findings indicate that MARCKS might play a role in regulating cell proliferation in the early phases of vascular remodeling. We have previously demonstrated that MARCKS knockdown *in vitro* inhibits VSMC growth, but only has a minimal effect on EC proliferation [7], making MARCKS a potential VSMC-specific molecular target for therapy to prevent restenosis. The mechanism by which MARCKS differentially affects proliferation in these two cell types is yet unknown.

MARCKS is an important regulator of cell proliferation in multiple cell types. MARCKS is a membrane-associated protein and a major substrate of protein kinase C (PKC). This signaling pathway is important in the regulation of cell cycle and proliferation [8,9]. MARCKS has also been implicated in the mitogen-activated kinase pathway [9,10]. Overexpression of MARCKS is associated with increased malignant potential in breast cancer [11], cholangiocarcinoma [12], hepatocellular carcinoma [13], and pancreatic cancer [14]. In contrast, down-regulation of MARCKS contributes to malignant cell proliferation and carcinogenesis in colon cancer [15], prostate cancer [16], glioma [17] and melanoma [18]. This seemingly conflicting evidence indicates that MARCKS-mediated regulation of proliferation is cell-type specific. These data correlate well with our previous report on the differential role of MARCKS in the proliferation of VSMCs and ECs [7].

The underlying mechanism for differential regulation of proliferation is yet unknown. MARCKS knockdown in *in vitro* is associated with a concomitant increase in p27^kip1^ expression [19]. However, in VSMCs, it is not known if the observed increase in p27^kip1^ is coincidental or responsible for the decrease in VSMC proliferation observed after MARCKS knockdown and whether it contributes to the differential regulation on EC proliferation. In the present investigation, we provide evidence that the increase in p27^kip1^ expression is directly responsible for the observed arrest in VSMC proliferation. We further demonstrate that MARCKS signaling differentially affects VSMC and EC proliferation through regulation of the kinase interacting with stathmin (KIS). And finally, we will provide evidence that MARCKS knockdown *in vivo* results in both decreased VSMC proliferation and an increased rate of re-establishment of an intact endothelium.

## Materials and Methods

### Animals

The animal research in the present investigation was approved by the University of Maryland Institutional Animal Care Committee (IACUC), protocol number 1110007. CL57/B6 wild-type and p27^kip1^ knockout mice were purchased from the Jackson Lab (Bar Harbor, ME). In all experiments the mice were 8–12 weeks old. Mice were maintained following the guidelines and protocols of the Animal Care and Use Committee of the University of Maryland School of Medicine.

### Cell culture

Human coronary artery vascular smooth muscle cells (CASMCs) and human coronary artery endothelial cells (CAECs) (Lonza, Walkersville, MD) were cultured according to the supplier’s instructions. The A7r5 rat aortic VSMC line was purchased and cultured in accordance with the supplier’s instructions (American Type Culture Collection, Manassas, VA). Cells between passage three and seven were used for experiments. Care was taken to seed cells at a density such that they would be sub-confluent after five days.

### Isolation and culture of primary murine aortic smooth muscle cells

Primary murine aortic smooth muscle cells were obtained from wild-type and p27^kip1^ knockout (p27^kip1^ -/-) mice (Jackson Lab, Bar Harbor, ME) as previously described [20]. Briefly, mice were anesthetized with inhaled 2% isoflurane and then euthanized by perfusion with phosphate buffered saline (PBS) through the left ventricle. A ventral incision was made to expose the aorta from the arch to its bifurcation. Under a dissecting microscope, the adventitia was dissected free, the aorta was isolated, and cut into square segments approximately 1 mm by 1 mm. The aorta tissue was digested with sterile 0.14% type II collagenase (Worthington Biochemical Corporation, Lakewood, NJ) in Dulbecco’s modified Eagle medium (DMEM) with 10% fetal bovine serum (FBS) for four hours at 37°C with 5% CO_2_ supply. The cells were then washed once in PBS, collected and cultured in DMEM supplemented with 10% FBS in 48-well plates for 5 days then the media was changed to standard smooth muscle cell media. Expression of smooth muscle α-actin was detected by immunofluorescence to confirm cell identity. Experiments were performed on these cells between passages two and four.

### siRNA and plasmid transfection

MARCKS siRNA (5’-GGU GCC CAG UUC UCC AAG AUU-3’), and non-targeting, control siRNA (5’-CGC ACC AGA ACA AAC ACA UU -3’) were purchased from Dharmacon (Lafayette, CO). The plasmids pCDNA3.1 KIS and pCDNA3.1 K54R KIS were kindly provided by Dr. Alexandre Maucuer (Howard Hughes Medical Institute, University of Massachusetts Medical School) [21]. Both siRNA and plasmids were transfected using the Dharma FECT-Duo transfection reagent (Thermo Scientific).

### Cell cycle analysis

Cells were treated with non-targeting, control siRNA or MARCKS siRNA for 24 hours and then split into new dishes at 3,300 cells/cm^2^ and cultured subconfluently with full medium. Cells were fixed with 70% ethanol at previously specified time points. Fixed cells were then treated with RNase and stained with Propridium iodide (PI). Cellular DNA content was measured with the BD Fortessa flow cytometer (BD Biosciences) as previously described to determine the proportion of cells in G_0_/G_1_, S, and G_2_/M phase [22,23].

### Western blot analysis

Protein expression in human CASMCs, human CAECs, murine aortic SMCs, and A7r5 cells was determined by Western blot analysis. The following antibodies were used in this investigation. Human smooth muscle cell KIS (catalog #SAB1300125), α-actin (catalog #2547), and β-actin (catalog #A5441) were purchased from Sigma (St. Louis, MO). Antibodies to cyclin D_1_, (catalog #2978), cyclin E_1_ (catalog #4129), Skp2 (catalog #2652), p27^kip1^ (catalog #3698), and proliferating cell nuclear antigen (PCNA) (Cat#2586) were obtained from Cell Signaling (Cell Signaling Tech., Danvers, MA). Antibodies to Phospho-Ser10-p27^kip1^ (catalog #346300) and phospho-Ser187-p27^kip1^ (catalog #717700), and pre-immuned rabbit IgG (Catalog #626111) were purchased from Invitrogen (Invitrogen, Grand Island, NY). These antibodies were used at the dilution that the manufacturer recommended for Western Blot analysis. The anti-rat KIS antibody was kindly provided by Dr. Alexandre Maucuer (Howard Hughes Medical Institute, University of Massachusetts Medical School). GAPDH or β-actin was used as loading controls. Protein levels were quantitated by performing densitometry using the Image Station 4000 MMPro (Carestream Health, Rochester, NY).

### Confocal microscopy

Cells were treated with control, non-targeting siRNA or MARCKS siRNA and then seeded onto fibronectin-coated coverslips and cultured in full medium. After 72 hours, cells were fixed with 4% paraformaldehyde and co-stained with anti-p27^kip1^ primary antibody and anti-MARCKS primary antibody (Cell Signaling catalog #5607) followed by staining with 4’,6-diamidino-2-phenylindole (DAPI) and FITC-conjugated or Alexa Fluor 546-conjugated secondary antibodies (Invitrogen, Carlsbad, CA) respectively. Stained cells were inspected under Zeiss 150 Duo laser confocal microscope (Carl Zeiss, Germany). Nuclear localization was defined as p27^kip1^ co-localizing with DAPI. Zeiss Zen imaging software was used to collect and analyze the image data. Experiments were performed in triplicate, and 50 cells were counted for each experiment. Significance of association was determined by the two-tailed Student’s *t*-test.

### Cell proliferation assay

Cellular proliferation was determined by the AlamarBlue assay as previously described [24]. Cells were plated at 3,300 cells/cm^2^ and cultured subconfluently with full medium for 24 hours. The cells remained subconfluent five days after plating. The cells were then incubated for 4 hours with the AlamarBlue reagent (Invitrogen, Carlsbad, CA). Fluorescence emission at 590 nm was determined with an excitation at 509 nm. Relative proliferation was determined by normalizing total fluorescence at a given time point to the fluorescence intensity determined by the first assay (time 0).

### Mouse aortic transmural mechanical injury and siRNA treatment

To create an *in vivo* system in which we could observe both the VSMC proliferative response to iatrogenic trauma and simultaneously assess re-endothelialization, we used a transmural mechanical injury of the infrarenal aorta. CL57/B6 mice were maintained and operated following the guidelines and protocols of the Animal Care and Use Committee of the University of Maryland School Of Medicine. At the time of surgery, mice were anesthetized with inhaled 2% isoflurane. A ventral laparotomy was made and the infrarenal aorta was exposed. The segment of the aorta from the left renal vein to the aortic bifurcation was injured. The crush injury was carried out through 30 serial crushes of 5-second duration with a cotton-tipped applicator. To ensure uniformity, a single vascular surgeon performed all injuries. This surgeon was blinded to the experimental condition of the mice. Immediately after injury, either 10 μM MARCKS siRNA or non-targeting siRNA suspended in 30% Pluronic F-127 gel (Sigma, St. Louis, MO) in PBS was applied to the exterior surface of the infrarenal aorta. After the gel solidified, the abdomen was closed and the animal was allowed to recover.

### mRNA quantification of a single aorta

Reverse transcription polymerase chain reaction (RT-PCR) was used to amplify and quantify mRNA expression in individual murine aortas. Total RNA was purified from each infrarenal aorta using the total RNA purification kit (Qiagen, Gaithersburg, MD) and RT-PCR was carried out using the Superscript III One-step RT-PCR system (Thermo Fisher, Waltham, MA) following the instructions from the manufacturer. Primers used for MARCKS amplification were: 5'-TTG TTG AAG AAG CCA GCA TGG G and 5'-CTG CCG TTG GCC TGC AGC TC; glyceraldehyde 3-phosphate dehydrogenase (GAPDH) primers were: 5'-CCT TCA TTG ACC TCA ACT AC and 5'-CCA AAG TTG TCA TGG ATG ACC. MARCKS mRNA expression was normalized to GAPDH expression.

### Immunohistochemistry

The experimental mice were euthanized at predetermined time points. The mice were anesthetized and perfused with 1.5% paraformaldehyde and 0.1% glutaraldehyde through the left ventricle accessed by a sternotomy. The infrarenal aorta was dissected from surrounding tissue then snap-frozen in optimal cutting temperature (OCT) compound (Sakura, Torrance, CA). Frozen sections were stained with hematoxylin and eosin (H&E) or immunostained with anti-MARCKS antibody (Cat# AB9298; Millipore, Billerica, MA) and anti-Ki-67 antibody (catalog #550609; BD, Franklin Lakes, NJ). The nucleus was counterstained with 4’,6-diamidino-2-phenylindole (DAPI) (Sigma, St. Louis, MO). Confocal microscopic images of the stained sections were acquired using a Zeiss 510 Duo laser confocal microscope (Carl Zeiss, Germany). Zeiss Zen imaging software was used to collect and analyze the image data.

### *In vivo* cell proliferation assay with 5-Bromo-2'-Deoxyuridine (BrdU) labeling

Mice were injected intraperitonally with BrdU (100 μg/g body weight) at 24 hours and again at 4 hours before harvesting the aorta. The infrarenal aorta was dissected and fixed as described above. Staining with anti-BrdU antibody (catalog #555627, BD Biosciences, Franklin Lakes, NJ) was performed as described previously [25]. Briefly, frozen sections were permeablized and treated with 50 U/ml DNase I at room temperature for 20 minutes to randomly and partially digest the chromosome DNA before the antibodies were applied. To quantify BrdU positive cells, digital photographs of the BrdU labeled aorta sections were analyzed using Zeiss Zen software (Carl Zeiss, Germany).

### *en face* immunostaining of aortic endothelium

*en face* preparation of the aorta was performed as described [26]. Briefly, mice were anaesthetized and perfusion fixed with 1% paraformaldehyde in PBS. The infrarenal aorta was dissected and then blocked with goat serum (5% donkey serum, 0.2% BSA, 0.3% TritonX-100 and 0.05% sodium azide in PBS) for 1 hour at room temperature. Whole-mount staining was performed using indicated primary antibodies for 16 hours at 4°C followed by washing using 0.3%-TritonX-100–PBS at room temperature, incubation with secondary antibodies for 16 hours, washing and post- fixing of the samples. The aortas were mounted in DAPI-Vectashield and fluorescent z-stack images were obtained using Zeiss 510 Duo.

### Evans Blue staining and quantification

Evans Blue dye was used to determine the extent of endothelium damage and re-endothelialization after mechanical aortic injury. At the time of euthanasia, the animals were perfused with 5 ml of 0.3% Evans Blue dye (Sigma, catalog #E2129) followed by 5 ml PBS at physiological pressure. The aorta was excised from the arch to the bifurcation then incised longitudinally to expose the intima and examined for Evans Blue staining. A quantification method was developed based on the Aoki protocol with minor modification [27]. Aortic tissue was weighed and homogenized in 50% trichloroacetic acid solution (500 ul/mg aorta tissue). Lysates were then centrifuged at 15,000 relative centrifuge force for 30 minutes at 4°C. The fluorescence (emission 610 nm/excitation 680 nm) of supernatant was measured in a microplate fluorescence reader (BioTek, Winooski, VT). The concentration of Evans Blue dye in the aorta extracts was calculated based on external standards.

### Statistical analysis

All experiments were performed in three independent replicates unless otherwise noted. Data are presented as means ± standard deviation. Statistical analysis was performed on STATA software (STATA Corporation, College Station, TX, USA). Significance of association was assessed using two-tail Student’s *t*-test, or Pierson’s chi-square test. A *p*-value less than 0.05 was considered statistically significant. All data are available online as Supporting Information found in the S1 Dataset.

## Results

### MARCKS knockdown arrests VSMC cell cycle progression at phase G_0_/G_1_

**Fig 1:**
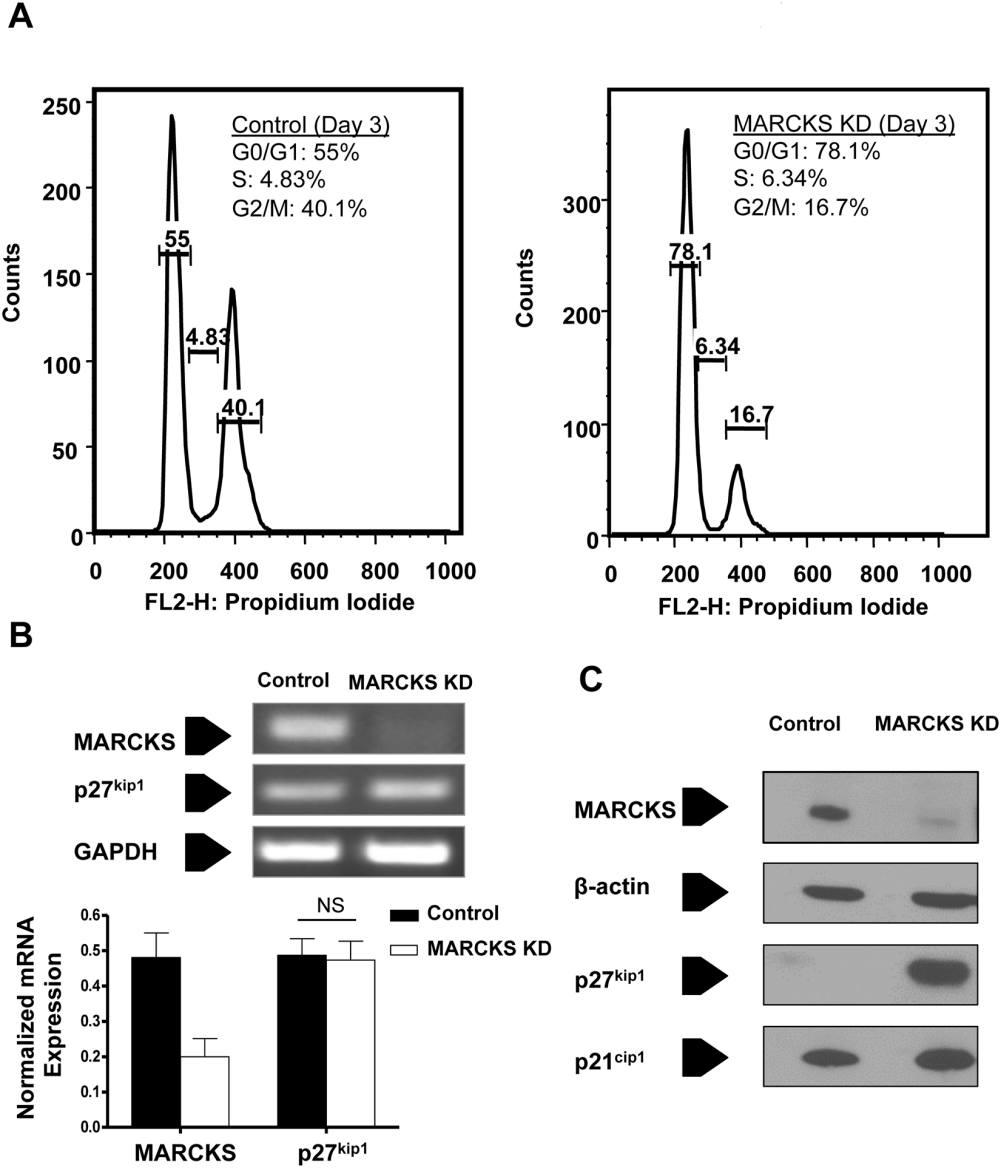
Cell cycle analysis after siRNA-mediated MARCKS knockdown in vascular smooth muscle cells. **A.** Cell cycle progression of human coronary artery smooth muscle cells (CASMCs) was determined by FACS analysis after treated with either 80 nM control, non-targeting siRNA, or MARCKS siRNA. MARCKS knockdown resulted in a significantly greater proportion of cells in phase G_0_/G_1_ compared to cells treated with control non-targeting siRNA, 78% compared to 55% respectively. MARCKS knockdown also resulted in fewer cells in phase G2/M than control, 17% and 40% respectively (Χ^2^ statistic 13.3, *p*<0.05). A representative histogram of the flow cytometry data from day three is presented. **B.** MARCKS mRNA knockdown was confirmed by RT-PCR. MARCKS knockdown had no effect on p27^kip1^ mRNA. **C.** MARCKS protein knockdown was confirmed by Western blot analysis. MARCKS knockdown had no effect on p21^cip1^ expression, but resulted in a large increase in p27^kip1^ protein levels.

CASMCs were assessed 1, 3, and 5 days after siRNA treatment for cell cycle progression using flow cytometry. A representative histogram of the data from day 3 is presented in Fig 1A. Three days after transfection, CASMCs treated with non-targeting siRNA resulted in 55% of cells in phase G_0_/G_1_, while treatment with MARCKS siRNA resulted in 78% of cells in phase G_0_/G_1_. Correspondingly, treatment with MARCKS siRNA resulted in significantly fewer cells in G_2_/M, 17%, compared to treatment with control siRNA, 40% (Χ^2^ statistic 13.3, *p*<0.05). Statistical significance was achieved at all time points and there was no significant apoptosis detected in either treatment condition (data not shown). RT-PCR analysis confirmed RNA knockdown after treatment with MARCKS siRNA (Fig 1B). Western blot analysis confirmed protein knockdown in cells treated with MARCKS siRNA (Fig 1C).

The cyclin dependent kinase inhibitors (CDKIs) p21^cip1^ and p27^kip1^ are important regulators of cell cycle progression from phase G1 to S [28,29]. MARCKS knockdown had no effect on p21^cip1^ expression, but resulted in a large increase in p27^kip1^ protein expression (Fig 1C). MARCKS knockdown had no effect on p27^kip1^ mRNA (Fig 1B).

### MARCKS knockdown does not affect cell proliferation in p27^kip1^ -/- VSMCs

**Fig 2:**
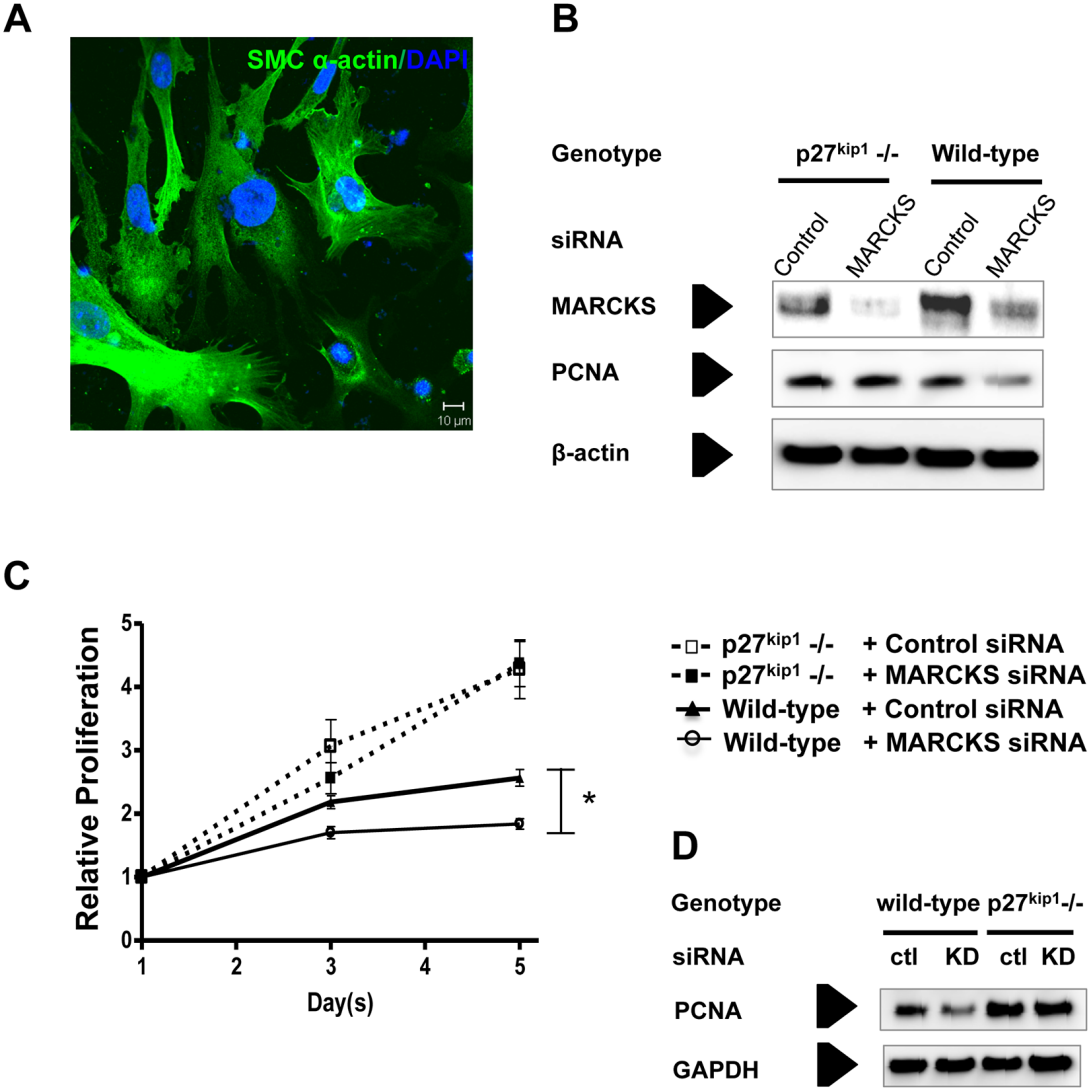
MARCKS knockdown does not affect proliferation of p27^kip1^ -/- vascular smooth muscle cells. **A.** Aortic smooth muscle cells were isolated from wild-type C57BL/6 or p27^kip1^-/- mice. Cells were stained with anti-smooth muscle α-actin to confirm smooth muscle cell type (scale bar = 10μm). **B.** Cells were treated with either non-targeting (Control) or MARCKS siRNA and protein expression was determined with Western Blot analysis to confirm MARCKS knockdown. **C.** Serial AlamarBlue proliferation assays were performed after siRNA treatment to determine relative cell proliferation. Cells were cultured at subconfluence and they remained subconfluent for the duration of the experiment. MARCKS knockdown significantly attenuated proliferation in aortic VSMCs from wild-type mice. As expected, VSMCs isolated from p27^kip1^ -/- mice proliferated significantly faster than the cells derived from wild-type mice. MARCKS knockdown had no effect on p27^kip1^-/- cell proliferation suggesting that MARCKS knockdown inhibits VSMC proliferation through a p27^kip1^-dependent mechanism. **D.** Proliferation was confirmed by assessing levels of proliferating cell nuclear antigen (PCNA). Statistical significance of association was determined by the two-tailed Student’s *t*-test. All experiments were performed in triplicate, * denotes *p*<0.05.

To confirm that the arrest of proliferation in MARCKS knockdown is p27^kip1^-dependent, we analyzed the effect of MARCKS knockdown in p27^kip1^-/- mouse VSMCs. Aortic VSMCs from p27^kip1^-/- and wild-type mice were harvested and cultured in DMEM and 10% FBS. Cells were stained with anti-smooth muscle α-actin to confirm VSMC identity (Fig 2A). The cells were then treated with either MARCKS siRNA or non-targeting siRNA. After siRNA transfection, MARCKS protein expression was significantly decreased in the knockdown group compared with the control group in both cell types (Fig 2B). Cell proliferation was determined by the AlmarBlue assay on days 1, 3, and 5 after transfection with siRNA. VSMCs derived from the p27^kip1^-/- mice had a baseline proliferation rate approximately twice as fast as VSMCs derived from wild-type mice. MARCKS knockdown attenuated proliferation of wild-type VSMCs compared to mice treated with non-targeting siRNA (Fig 2C, *p*<0.05, n = 3). However, MARCKS knockdown had no significant effect on the proliferation of VSMCs derived from p27^kip1^-/- mice (Fig 2C). Proliferation was confirmed by assessing levels of proliferating cell nuclear antigen (PCNA) (Fig 2D).

### MARCKS knockdown inhibits phosphorylation of p27^kip1^ at serine 10

**Fig 3:**
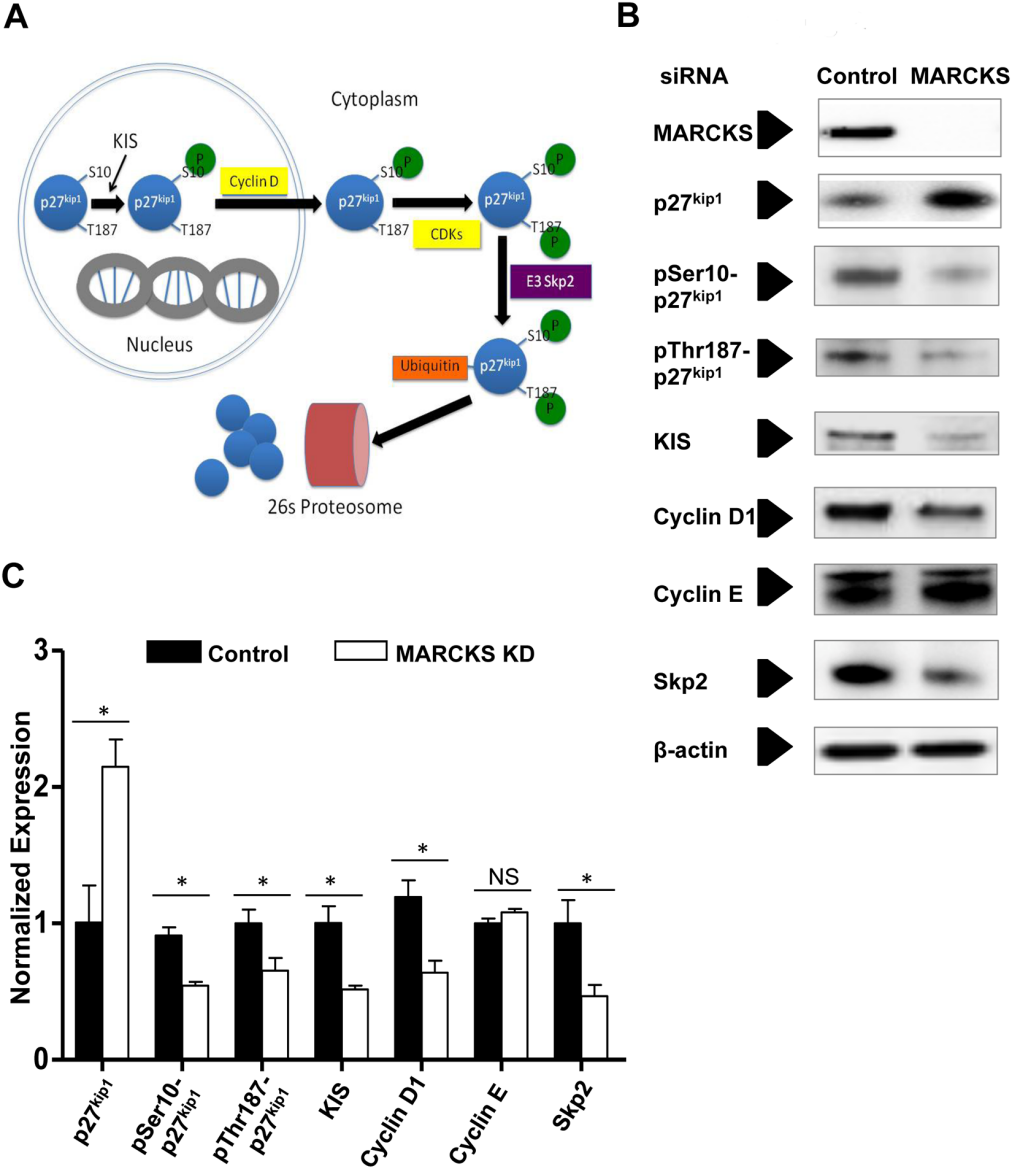
MARCKS knockdown decreases p27^kip1^, pSer10-p27^kip1^, KIS, cyclin D1, and ubiquitin ligase E3 Skp2, but does not affect cyclin E1 protein expression. **A.** The cyclin-dependent kinase inhibitor p27^kip1^ protein expression is regulated in a multistep process by degradation by the 26s proteasome. **B.** Human coronary artery smooth muscle cells (CASMCs) were treated with MARCKS siRNA or non-targeting siRNA (Control). Protein expression was determined by Western Blot analysis. **C.** Protein expression was normalized to β-actin and compared with densitometry. Expression of pSer10-p27^kip1^, pThr187-p27^kip1^, KIS, cyclin D1, and the E3 ubiquitin protein ligase all decreased significantly with MARCKS knockdown. Only Cylcin E did not change significantly as a result of MARCKS knockdown. KIS and pSer10-p27^kip1^ had the greatest decrease in protein expression as a result of MARCKS knockdown. All experiments were performed in triplicate. Statistical significance was determined by the two-tailed Student’s *t*-test. * denotes *p*<0.05.

Protein levels of p27^kip1^ fluctuate during the cell cycle, with the highest level occurring at G_0_/G_1_ and subsequently decreases through proteolysis allowing cells to progress from phase G_0_/G_1_ to S [30]. Canonically, p27^kip1^ degradation is a multistep process involving several key regulators of p27^kip1^ phosphorylation: first, p27^kip1^ is phosphorylated at serine 10 (pSer10-p27^kip1^) by kinase interacting with stathmin (KIS) [31], which allows its export from the nucleus in association with cyclin D. In the cytoplasm p27^kip1^ is further phosphorylated at threonine 187 (pThr187-p27^kip1^) by CDK2, then ubiquinated by the ubiquitin ligase E3 Skp2, and ultimately degraded by the 26s proteasome (Fig 3A) [32]. After treatment of human CASMCs with MARCKS siRNA, there was a significant decrease in the protein expression of pSer10-p27^kip1^, pThr187-p27^kip1^, and ubiquitin ligase E3 Skp2 (Fig 3B and 3C, * denotes *p*<0.05, n = 3). Additionally, there was a significant decrease (60.7%±5.7%) in KIS expression. No change was observed in expression of Cyclin E (Fig 3B, *p* = NS). The change in pSer10-p27^kip1^ and KIS expression were greatest in magnitude, a 61.3% and 60.7% decrease respectively, among intermediate products and enzymes studied (Fig 3C).

### MARCKS knockdown results in nuclear trapping of p27^kip1^

Phosphorylation at serine 10 is necessary for p27^kip1^ export from the nucleus and is the most proximal step in the process of p27^kip1^ degradation. Because VSMC arrests proliferation at phase G_1_ after MARCKS knockdown, and causes a decrease in pSer10-p27^kip1^, we hypothesized MARCKS knockdown would result in an accumulation of p27^kip1^ in the nucleus. Subconfluent CASMCs were transfected with either control or MARCKS siRNA for three days.

**Fig 4:**
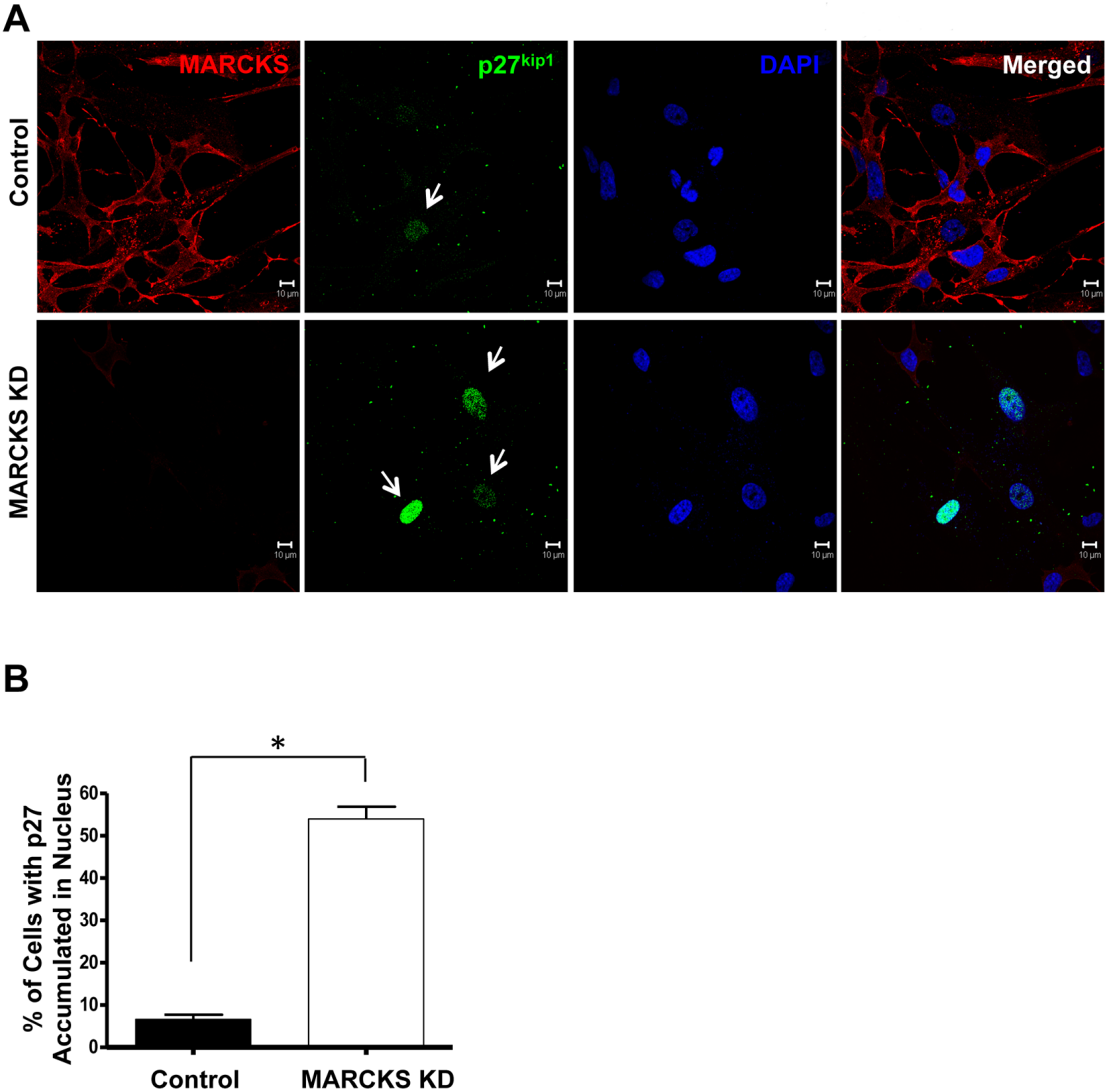
MARCKS knockdown results in p27^kip1^ nuclear trapping. **A.** Human coronary artery smooth muscle cells (CASMCs) were transfected with non-targeting, control siRNA, or MARCKS siRNA. MARCKS knockdown was associated with both increased total p27^kip1^ and nuclear trapping of p27^kip1^. Nuclei were counterstained with DAPI. Scale bar = 10μm. **B.** MARCKS knockdown resulted in 54.0±5.1% of cells with nuclear localization of p27^kip1^ accumulation in nucleus while only p27^kip1^ localized to the nucleus in only 6.7±1.9% cells in the control group. Experiments were performed in triplicate and 150 cells were counted for each treatment. Statistical significance was determined by the two-tailed Student’s *t*-test. * denotes *p*<0.05.

MARCKS knockdown resulted in increased nuclear staining for p27^kip1^ as assessed by laser confocal microscopy (Fig 4A). Control cells had scant staining of p27^kip1^ in the cytoplasm with minimal nuclear localization. Nuclear localization was confirmed by co-staining with DAPI. MARCKS knockdown resulted in 54.0±5.1% of cells with nuclear localization of p27^kip1^ accumulation in nucleus while only p27^kip1^ localized to the nucleus in only 6.7±1.9% cells in the control group. (Fig 4B, *p*<0.05).

### MARCKS knockdown-induced nuclear trapping of p27^kip1^ is released by overexpression of KIS

**Fig 5:**
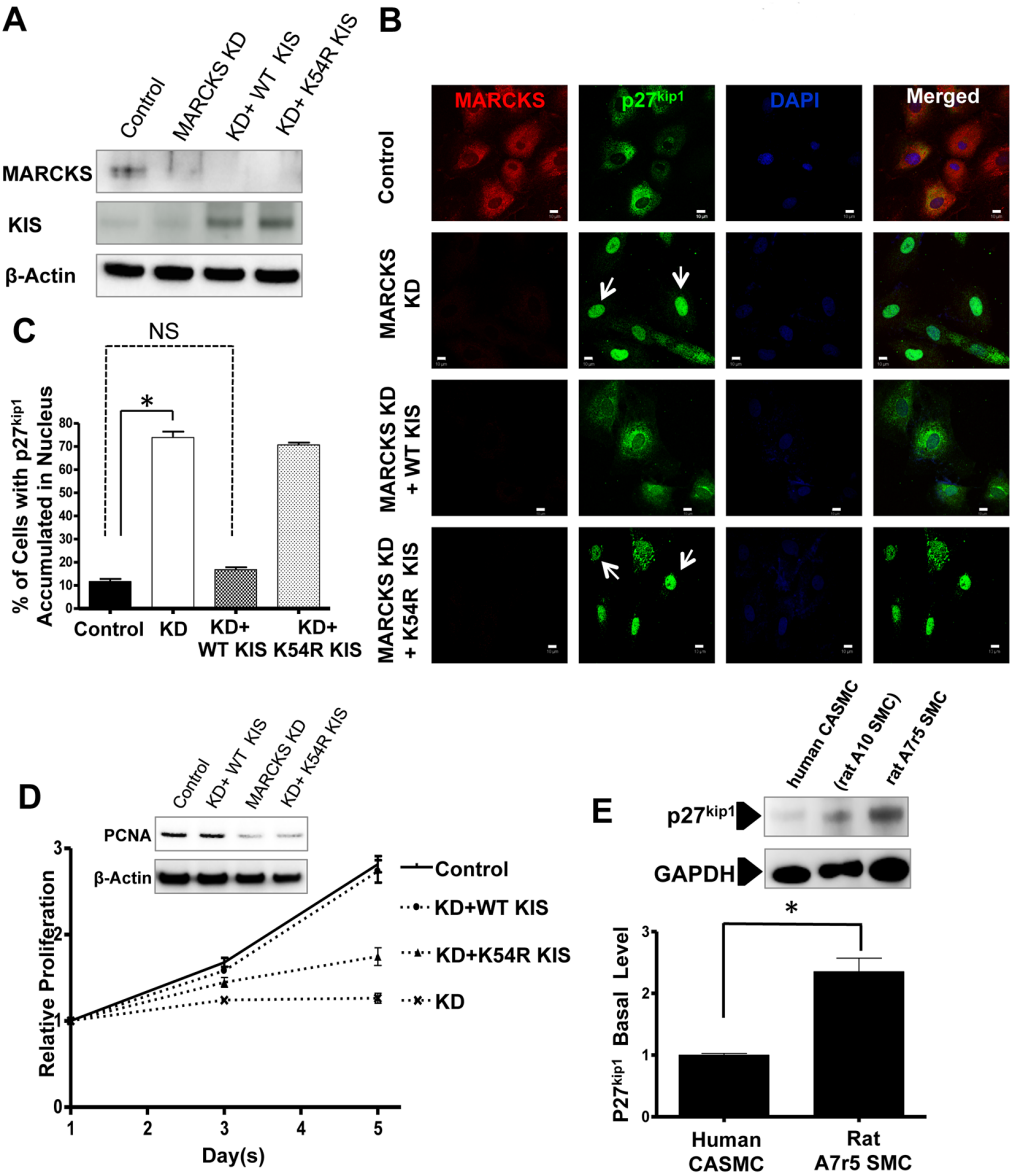
MARCKS knockdown induced p27^kip1^ nuclear trapping is released by co-transfection with wild-type kinase interacting with stathmin (KIS). **A.** Rat aortic vascular smooth muscle A7r5 cells were transfected with either non-targeting, control siRNA or MARCKS siRNA and co-transfected with DNA plasmids expressing wild-type KIS (WT KIS), or kinase-dead mutant KIS (K54R KIS). MARCKS siRNA knockdown and expression of KIS plasmids were confirmed by Western blot analysis. **B.** Co-transfection with wild-type KIS released the nuclear trapping of p27^kip1^ (white arrows) in MARCKS knockdown cells, but kinase-dead KIS (K45R KIS) did not. **C.** A total of 150 cells were scored in each treatment allowing for quantitative analysis (white arrows in Fig 5B indicate examples of cells counted as with phenotype of p27^kip1^nuclear accumulation). Nuclear trapping of p27^kip1^ after MARCKS knockdown was released by co-transfection with wild-type KIS but not kinase dead KIS (K45R). Scale bar = 10 μm, * denotes p<0.05. **D.** Vascular smooth muscle cells were cultured at subconfluence and they remained subconfluent for the duration of the experiment. MARCKS knockdown arrested vascular smooth muscle cell proliferation. This proliferation defect was rescued by co-transfection with wide-type KIS, but not kinase-dead KIS. Proliferation was confirmed by assessing levels of proliferating cell nuclear antigen (PCNA). **E.** Compared to CAMCs, the A7r5 cells express higher constitutive levels of p27^kip1^. Experiments were performed in triplicate. Statistical significance was determined by the two-tail Student’s *t*-test. * denotes *p*<0.05, NS denotes not significant.

KIS (kinase interacting with stathmin) is a critical regulator of cell cycle progress, which catalyzes p27^kip1^ phosphorylation at serine 10 and nuclear exportation during the transition from phase G0/G1 to S. A single amino acid mutation, K54R, abolishes KIS kinase function [31,33]. Both KIS and pSer10-p27^kip1^ protein levels were significantly decreased by MARCKS knockdown. A7r5 rat aortic smooth muscle cells were transfected with both MARCKS siRNA and the plasmid encoding KIS (WT) or the plasmid encoding kinase dead KIS (K54R KIS). The A7r5 cell line was used in this rescue experiment making use of its much higher transfection efficiency for foreign plasmid DNA. MARCKS was only detectable in cells not transfected with MARCKS siRNA. KIS was expressed at very low levels in both control cells and MARCKS siRNA treated cells. KIS was expressed in much higher levels in cells co-transfected with MARCKS siRNA and either the WT KIS plasmid or the K45R KIS plasmid as the antibody detects both WT KIS and K45R KIS (Fig 5A). Similar to CASMCs, MARCKS knockdown resulted in p27^kip1^ accumulation in the nucleus of the A7r5 cells (white arrows, Fig 5B). Nuclear trapping of p27^kip1^ was found in 73.9%±4.4% of MARCKS siRNA-treated cells whereas treatment with non-targeting siRNA resulted in nuclear trapping of p27^kip1^ in only 11.7%±1.9% of cells (white arrows Fig 5B and 5C, *p*<0.05). Co-transfection with the WT KIS plasmids and MARCKS siRNA resulted in increased p27^kip1^ expression compared to control cells, but p27^kip1^ did not accumulate in the nucleus. Co-transfection with both MARCKS siRNA and the WT KIS plasmid resulted in only 16.5%±1.7% of cells with p27^kip1^ accumulated in the nucleus. The proportion of cells with p27^kip1^ trapped in the nucleus was the same in control cells and cells cotransfected with MARCKS siRNA and WT KIS (Fig 5B and 5C, *p* = not significant). However, co-transfection of K54R KIS (kinase dead KIS) with MARCKS siRNA failed to reverse the effect of MARCKS knockdown with p27^kip1^ localized to the nucleus in 69%±1.7% of cells. A similar proportion of cells had p27^kip1^ trapped in the nucleus compared with MARCKS siRNA alone. In A7R5 cells, MARCKS knockdown results in attenuated cell proliferation. Proliferation was restored to normal by co-transfection with wild-type KIS, but not kinase dead KIS. Proliferation was confirmed by assessing levels of PCNA (Fig 5D). Thus, increasing KIS expression released the MARCKS-knockdown induced nuclear trapping of p27^kip1^ and rescued the VSMCs proliferation defects. Compared to CAMCs, the A7r5 cells express higher constituative levels of p27^kip1^(Fig 5E). This difference explains why the A7r5 cells stain for p27^kip1^ in the cytoplasm whereas the CASMCs (Fig 4) had only scant staining.

### MARCKS knockdown in human coronary artery ECs increases KIS expression and promotes EC proliferation

**Fig 6:**
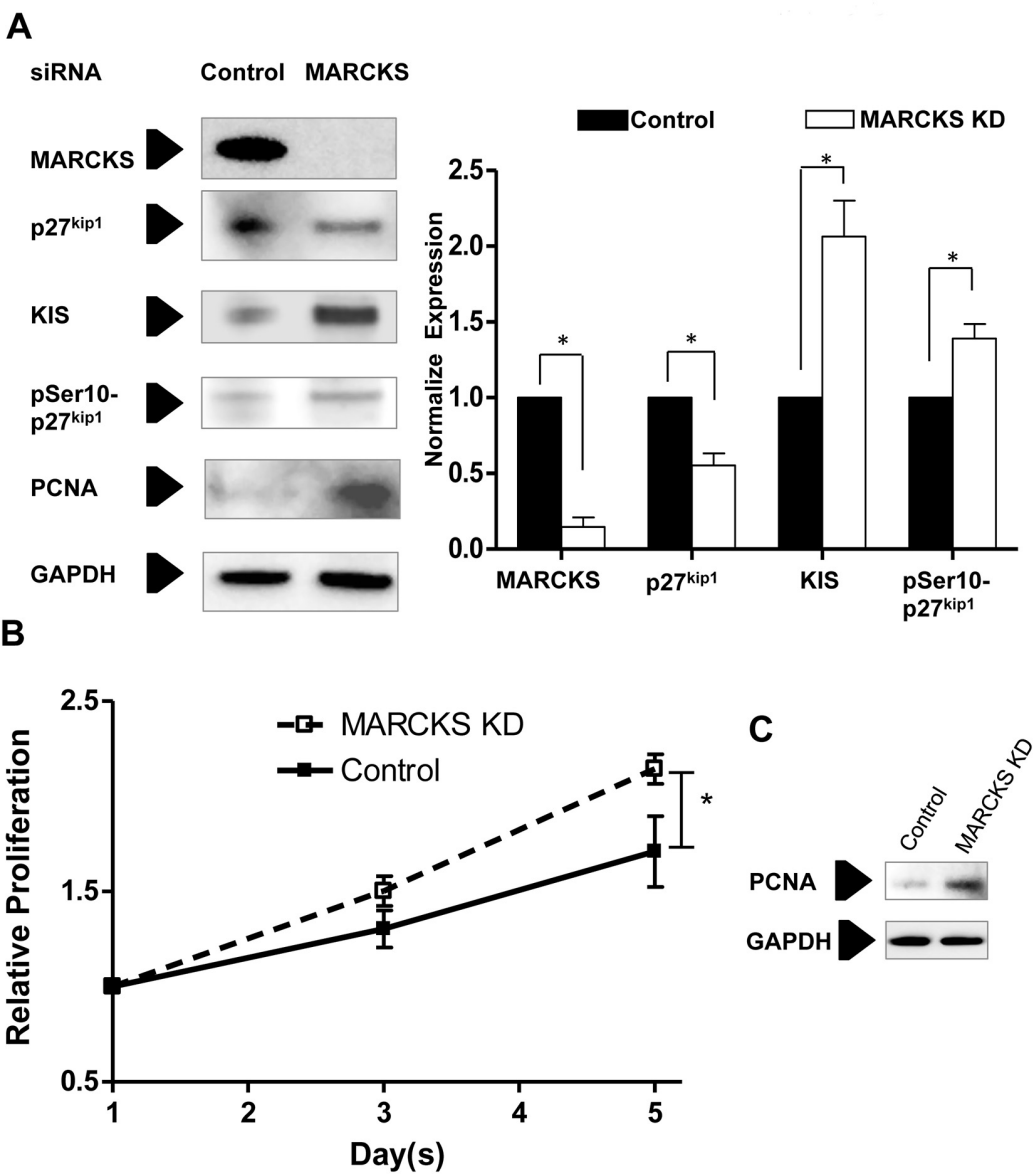
MARCKS knockdown increases KIS expression and cell proliferation in endothelial cells. **A.** Human coronary artery ECs were treated with 20 nM non-targeting, control or MARCKS siRNA. Four days after transfection, MARCKS knockdown increased both KIS and pSer10-p27^kip1^ protein expression. **B.** Endothelial cells were cultured at subconfluence and they remained subconfluent for the duration of the experiment. MARCKS knockdown also resulted in 25±6% (*p*<0.05) more proliferation at five days after transfection. **C.** Proliferation was confirmed by assessing levels of proliferating cell nuclear antigen (PCNA). Statistical significance was determined by the two-tailed Student’s *t*-test. * denotes *p*<0.05.

In contrast to observations in VSMCs, siRNA-mediated MARCKS knockdown resulted in a significant increase of KIS expression in human CAECs. MARCKS knockdown also resulted in increased pSer10-p27^kip1^ and decreased levels of p27^kip1^ in CAECs (Fig 6A). Furthermore, in subconfluently cultured CAECs, MARCKS knockdown increased EC proliferation. Five days after MARCKS siRNA treatment, EC proliferated 25±6% (*p*<0.05) faster than EC treated with non-targeting siRNA (Fig 6B). Proliferation was confirmed by assessing levels of PCNA (Fig 6C).

### MARCKS protein expression is increased in response to mechanical vascular injury *in vivo*

**Fig 7:**
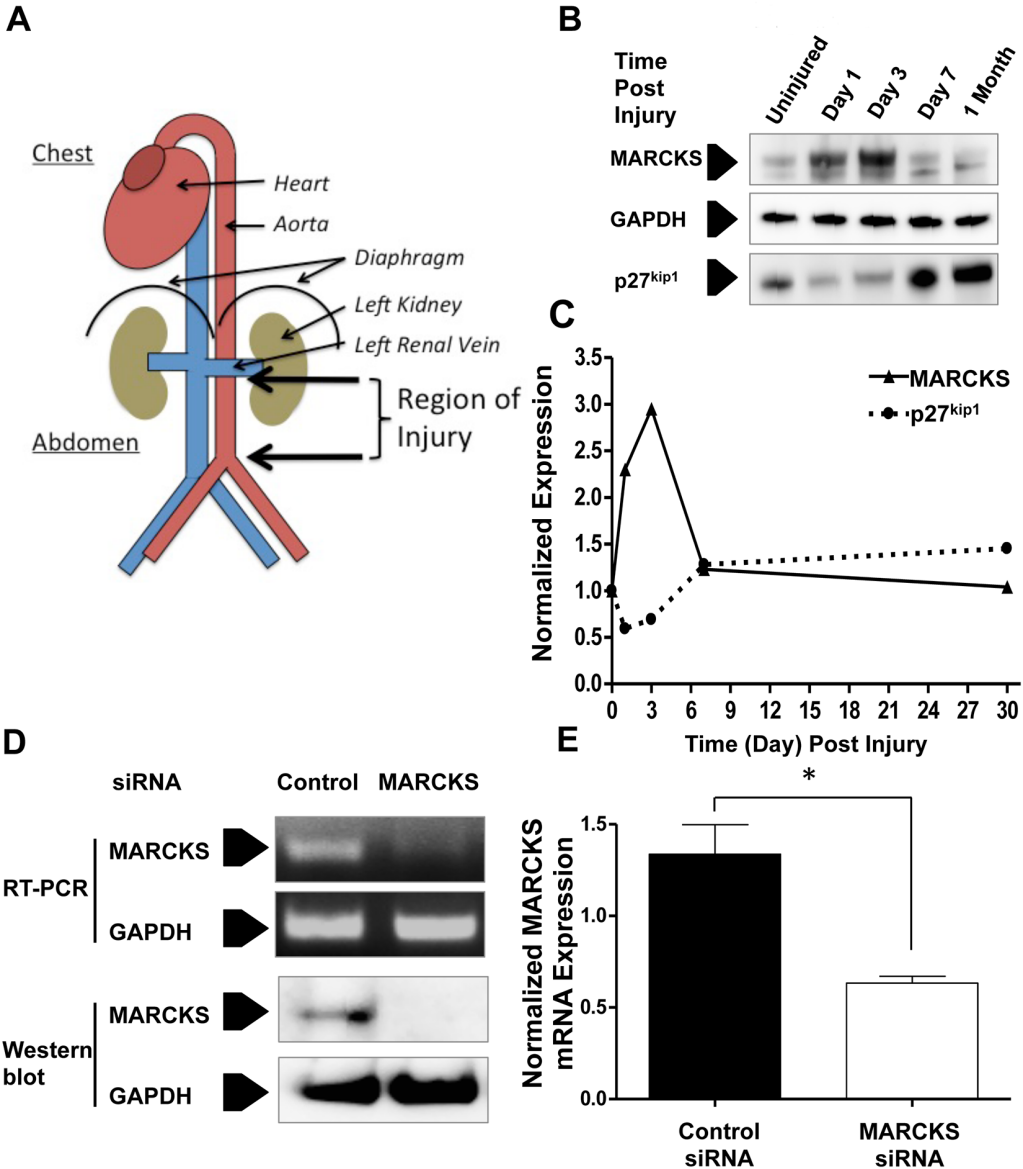
MARCKS protein expression is increased in the mouse aorta after stimulation by transmural mechanical injury. **A. The infrarenal mouse aorta was exposed to blunt transmural injury B.** The injury resulted in a 344% increase of MARCKS expression after three days. **C.** MARCKS expression returned to baseline one month after injury. The mechanical injury also resulted in a decrease in total p27^kip1^ expression in the injured aortas, which also returned to baseline after 30 days. **D.** At the time of surgery, the injured infrarenal aorta was treated with 10 μM MARCKS siRNA or 10 μM non-targeting, control siRNA suspended in 30% Pluronic F-127 gel. The animals were euthanized three days after surgery and knockdown of both mRNA and protein was assessed. MARCKS protein was not detected three days after transfection. **E.** Three days after injury MARCKS siRNA significantly decreased MARCKS mRNA by 42±15%. Statistical significance was determined by the two-tailed Student’s *t*-test. *denotes *p*<0.05, n = 6.

We examined MARCKS expression in response to a mechanical injury in the mouse infrarenal aorta (Fig 7A). MARCKS protein expression increased almost three-fold at 24-hours after injury (Fig 7B). Protein expression reached a maximum (344% of baseline) three days after injury and returned to baseline at one month after the injury. p27^kip1^ protein expression decreased by 71% one day after injury. Protein expression of p27^kip1^ returned to baseline seven days after injury (Fig 7C).

Overexpression of MARCKS was abrogated by treatment with MARCKS siRNA at the time of injury. Three days after the crush injury, injured aortas treated with MARCKS siRNA demonstrated a 42±15% (*p*<0.05, n = 5) decrease in MARCKS mRNA expression compared with aortas treated with non-targeting siRNA (Fig 7D and 7E). Treatment with MARCKS siRNA also reduced MARCKS protein expression *in vivo* confirmed with Western blot analysis of the total lysis of aortic tissues (Fig 7D).

### *in vivo* MARCKS knockdown decreases VSMC proliferation and prevents vascular remodeling after aorta crush injury

**Fig 8:**
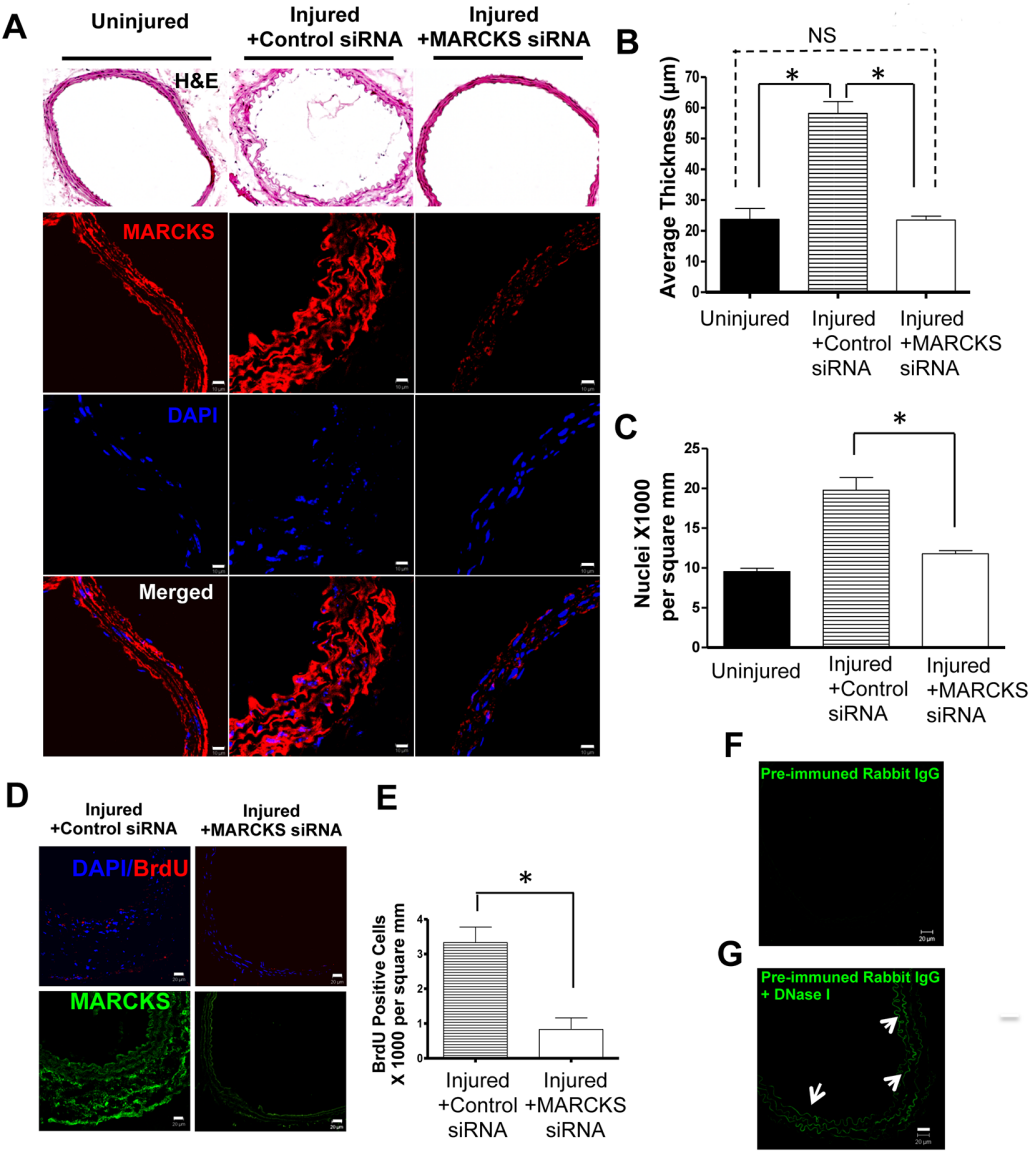
*in vivo* MARCKS knockdown decreased VSMC proliferation after transmural aortic injury. The murine infrarenal aorta was subjected to mechanical injury. **A.** Topical application of MARCKS siRNA in a 30% pluronic gel suspension at the time of injury resulted in decreased vascular remodeling as determined by wall thickness as shown in H&E staining and fluorescent immunostaining. **B.** Topical treatment with MARCKS siRNA at the time of injury prevented vessel wall thickening. **C.** The decreased wall thickness was due to a decrease in VSMC proliferation as determined by staining with DAPI. Cell proliferation was quantified by measuring the number of DAPI stained nuclei per mm^2^ (p<0.05). **D.** Additionally both BrdU staining and Ki-67 staining (data not shown) confirmed that there were fewer proliferating cells in the media after treatment with MARCKS siRNA. Scale bar = 10 μm. **E**. MARCKS knockdown resulted in significantly fewer cells per mm^2^ that stained poisitive for BrdU compared to aortas treated with non-targeting, control siRNA. Statistical significance was determined by the two-tailed Student’s *t*-test. *denotes *p*<0.05. **F**. Sections of the aortic wall had little staining with pre-immuned rabbit IgG. **G**. Treatment with DNase I resulted in increased non-specific staining of the aorta. The aortas in Figure A were not pre-treated with DNase I, however the sections in Figure D were treated with DNase I which explains the increase in background signal in Figure D.

The mouse transmural aortic injury used in the current study stimulates vascular smooth muscle cells and disrupts endothelial integrity. Therefore, this model provides an optimum *in vivo* condition to study the interaction and pathological reaction of vascular EC and VSMC in response to vascular injury simultaneously. The morphological change induced by the transmural crush injury was discernible at three days after injury. A high degree of MARCKS knockdown was achieved through topical application of siRNA (Fig 8A). The crush injury resulted in a 146%±99% increase in the thickness of the aorta wall three days after injury. However, knocking down MARCKS at the time of aortic injury, prevented wall thickening, a 1.0%±0.93% decrease in wall thickness was observed, compared with either no siRNA treatment or treatment with non-targeting siRNA (Fig 8B, *p< 0.05, n = 3).

To quantify VSMC proliferation after aorta injury, 5 μm thick frozen sections were prepared at two different locations along the treated aorta 100 μm apart and then stained for DAPI, BrdU, or Ki-67. MARCKS knockdown resulted in significantly decreased proliferation as quantified wtih DAPI (Fig 8C), BrdU (Fig 8D and 8E) and Ki-67 staining (data not shown).

In Fig 8A, there is little signal detected after MARCKS knockdown, however, there is obvious staining present after MARCKS knockdown in Fig 8D. To perform the BrdU assay, the sections were treated with DNase I. In sections not treated with DNase I, there is little non-specific staining with pre-immuned rabbit IgG (Fig 8F). Treatment with DNase I resulted in non-specific staining of the elastic lamina (Fig 8G). Increased non-specific staining after treatement DNase I is the reason why there is increased background staining in Fig 8D compared to Fig 8A.

### Mechanical transmural aortic injury disrupts the endothelium

**Fig 9:**
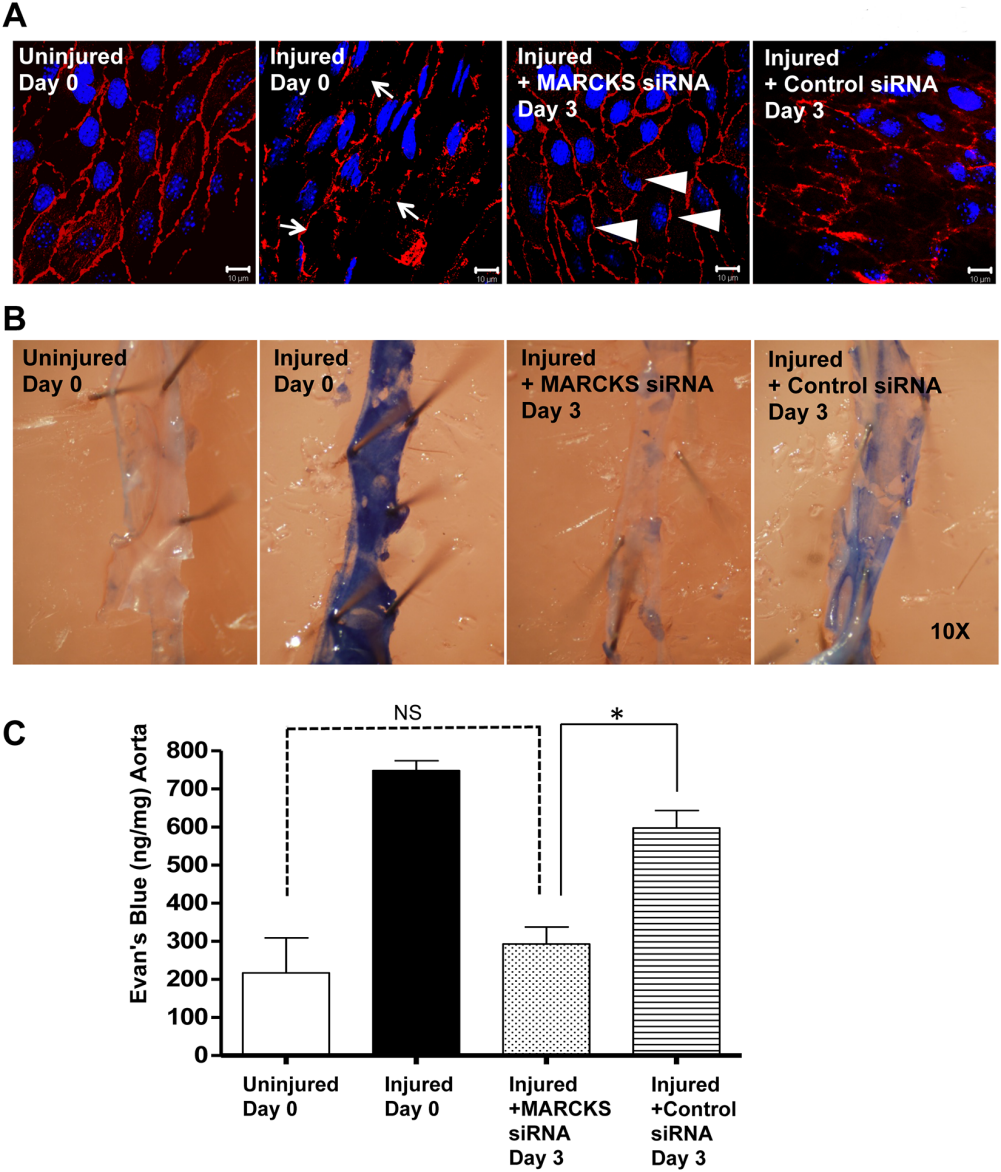
MARCKS knockdown increased endothelial integrity after aortic transmural crush injury. The mouse infrarenal aorta was exposed and subjected to crush injury as described previously. Upon injury, the aorta was treated topically with either MARCKS siRNA or non-targeting siRNA (Control) suspended in 30% pluronic gel. **A.** *en face* prepared aortas were stained with CD31 (red) and DAPI. The crush injury resulted in fewer nuclei in the vessel wall (white arrows). Treatment with MARCKS siRNA resulted in more nuclei (white triangles) in the aortic wall three days after injury compared to aortas treated with control siRNA. Scale bar = 10 μm. **B.** At the time of euthanasia, animals were perfused with 0.3% Evans Blue dye. This dye is excluded from aortas with an intact endothelium and thus there is the minimal staining in uninjured aortas. After the crush injury, aortas with damaged endothelium bind Evans Blue dye (magnificantion = 10x). **C.** Analysis of Evans Blue staining demonstrated increased endothelial integrity in injured aortas treated with MARCKS siRNA compared to injured aortas treated with non-targeting siRNA. Three days after injury, treatment with MARCKS siRNA resulted in significantly less Evans Blue dye staining than treatment with non-targeting siRNA (294.4 ± 78.8 ng/mg of MARCKS siRNA treated aortas versus 596.9 ± 77.6 ng/mg of control siRNA treated aortas. Statistical significance was determined by the two-tail Student’s *t*-test. * denotes *p*<0.05, NS denotes not significant

MARCKS knockdown increased re-endothelialization after aortic transmural crush injury. At the time of euthanasia, an enface preparation was made for each experimental condition. *En face* prepared aortas were stained with CD31 (red) and DAPI (Fig 9A). Aortic transmural crush injury severely injured the aorta endothelium and caused partial denudation immediately after injury. The crush injury resulted in fewer nuclei in the vessel wall (white arrows). Treatment with MARCKS siRNA resulted in more nuclei (white triangles) in the aortic wall three days after injury compared to aortas treated with control siRNA. Scale bar = 10 μm (Fig 9A).

### *in vivo* MARCKS knockdown decreases time to restoration of an intact endothelium

The mouse aortic transmural mechanical injury model allows quantification of the reestablishment of an intact endothelium after vascular mechanical injury. In this model, aortic injury results in denudation of the endothelium. Damaged endothelium allows staining with Evans Blue dye where as an intact endothelium does not permit staining with the dye. At the time of injury, the infrarenal aorta was treated with either MARCKS siRNA or non-targeting siRNA. Immediately after injury, the infrarenal aorta stained dark blue regardless of which siRNA was applied (Fig 9B). Three days after injury, treatment with MARCKS siRNA resulted in significantly less Evans Blue dye staining than treatment with non-targeting siRNA (294.4 ± 78.8 ng/mg of MARCKS siRNA treated aortas versus 596.9 ± 77.6 ng/mg of control siRNA treated aortas, *p*<0.05, Fig 9C). Seven days after injury both treatment conditions had regenerated an intact endothelium and the staining pattern resembled an uninjured aorta (data not shown).

## Discussion

This is the first report that MARCKS signaling differentially affects VSMC and EC proliferation through a p27^kip1^, KIS-dependent mechanism. First, MARCKS knockdown prevented cell cycle progression at phase G_0_/G_1_. Second, MARCKS knockdown resulted in increased protein level of the CDKI p27^kip1^. MARCKS knockdown had no effect on proliferation in p27^kip1^ -/- VSMCs. This finding affirms that MARCKS knockdown inhibits VSMC proliferation through a p27^kip1^-dependent mechanism. MARCKS knockdown results in trapping of p27^kip1^ in the nucleus likely a result of decreased p27^kip1^ phosphorylation at serine 10. Consistent with this observation, KIS protein level was significantly decreased by MARCKS knockdown in VSMCs. Overexpression of wild-type KIS in MARCKS-deficient cells released the nuclear trapping of p27^kip1^ and restored normal proliferation. Surprisingly, we found MARCKS knockdown did not decrease KIS expression in EC. On the contrary, MARCKS knockdown results in increased KIS expression and potentiated EC proliferation. These observations are consistent with our *in vivo* data demonstrating that MARCKS knockdown decreases medial thickening and results in earlier reestablishment of an intact endothelium. The differential effect of MARCKS knockdown on VSMC and EC proliferation makes MARCKS a well-suited target for the prevention of intimal hyperplasia after arterial interventions.

Progression of the cell cycle from G_0_/G_1_ to S is tightly controlled by p27^kip1^ [34,35]. The efficacy of p27^kip1^ maintaining G_0_/G_1_ is critical for VSMCs, since cell transition from G_0_/G_1_ to S results in increased proliferation and represents one of the earliest events in vascular proliferative diseases. MARCKS knockdown decreases p27^kip1^ phosphorylation at serine 10 and results in nuclear accumulation of p27^kip1^. These data provide a plausible mechanism for the regulation of VSMC G_0_/G_1_ to S transition through MARCKS. The efficacy of p27^kip1^ in maintaining phase G_0_/G_1_ is achieved by both the quantity of protein and localization in the nucleus where it binds to cyclin-dependent kinases (CDKs) and inhibits their function, preventing cell cycle progression. Our data show that p27^kip1^ mRNA is constitutively expressed in VSMCs which confirms other reports [36], and is not affected by MARCKS knockdown. Regulation of p27^kip1^ by MARCKS occurs at the post-transcriptional level. Canonically, p27^kip1^ protein expression is thought to be regulated through ubiquitin-mediated proteolytic processing [37], however, it is conceivable that any step in this pathway can be regulated affecting p27^kip1^ degradation.

MARCKS knockdown resulted in the change in expression of multiple kinases, enzymes, and their substrates in the p27^kip1^ pathway. However, pSer10-p27^kip1^ was down regulated to a greater degree than any other enzyme or metabolite (Fig 3). Phosphorylation of p27^kip1^ in nucleus is the most proximate step in the pathway, making it a candidate for a rate-limiting step in the entire biochemical pathway of p27^kip1^ degradation. MARCKS knockdown resulted in p27^kip1^ trapping in the nucleus. Nuclear trapping was released by co-transfection with wild-type KIS, as was the inhibition of proliferation by MARCKS.

The current study is the first report implicating MARCKS as a key upstream regulator for differential KIS expression in VSMCs and ECs. MARCKS knockdown caused differential expression of KIS in VSMCs and ECs, resulting in an arrest of proliferation in VSMCs but increased cellular proliferation in ECs. MARCKS knockdown *in vivo* in the aorta inhibits VSMC proliferation after stimulation by mechanical injury. On the surface, these results differ from those of Dr. Nabel and colleagues of KIS knockout mice, which developed a more robust hyperplastic response to injury than control mice. Whereas, our data suggests that decreased KIS expression would inhibit VSMC proliferation. KIS is a complex enzyme that regulates cell proliferation through two competing mechanisms. First, it catalyzes the phosphorylation of p27^kip1^ at serine 10 as outlined in this investigation. Second, it exerts a destabilizing effect on cytoplasmic tubulin. This effect favors increased proliferation [38]. We modulated KIS expression by knocking down MARCKS, one of its putative upstream effectors, as opposed to performing the experiments in a KIS knockout mouse. The differential regulation of KIS by MARCKS in ECs and VSMCs and the effect of KIS on proliferation is an intriguing area for future research.

This murine model of vascular proliferation is not as widely used as other models such as the carotid ligation or the femoral wire injury model. The murine carotid ligation model is a flow-mediated model of vascular proliferation in which the endothelium is left undisturbed. Obviously this model would not allow us to quantitate endothelial recovery. The femoral wire injury model denudes the endothelium and generates a proliferative response in the medial VSMCs. Our *in vitro* data demonstrated a 25% increase in EC proliferation as a result of MARCKS knockdown. Although Evans blue staining is possible in the femoral artery, the tissue mass of this specimen is prohibitively small and we did not believe that Evans blue staining of this artery would allow us to observe the anticipated modest effect of MARCKS knockdown on restoration of the endothelium.

The murine aorta is a thicker vessel than the carotid or femoral arteries, potentially making it a more clinically relevant model. Crush injury has been used to generate proliferative lesions in dogs [39], pigs [40,41], and in human saphenous vein in culture [42]. In addition to the more widely used carotid ligation and femoral wire injury models, electrical stimulation, placement of an external arterial cuff, air dry-induced injury, and chemical-induced injury have all been used in mouse models to initiate the formation of intimal hyperplasia [43]. The aortic crush model resulted in a robust, consistent proliferative response in the medial smooth muscle cells as determined by quantification of DAPI stained nuclei, BrdU staining, and Ki-67 staining. The proliferative VSMCs in these vessels also resulted in consistent vessel wall thickening.

Logically, current treatments to prevent vascular proliferation diseases have used antiproliferative agents such as sirolimus, mTOR inhibitors (mammalian target of rapamycin) and paclitaxel. Unfortunately, these antiproliferative agents are not VSMC specific, and prevent both VSMC and EC proliferation. Although they prevent neointima formation, they also prevent re-endothelialization resulting in a chronic thrombogenic surface in the treated vessel, which can result in life-threatening *in situ* thrombosis [44]. An ideal treatment would arrest VSMC proliferation long enough for the inflammatory stimulus to dissipate and the endothelium to regenerate. MARCKS knockdown increased EC proliferation *in vitro* and re-endothelialization *in vivo*. Interestingly, a p27^kip1^ mutation has been recently described in humans. This mutation renders p27^kip1^ non-functional and is associated with much greater rates of both in-stent restenosis and intimal hyperplasia of vein grafts than in the general population [45]. This clinic data supports the importance of p27^kip1^ and MARCKS as translational targets for restenosis therapy.

In summary, this is the first report of the mechanism through which MARCKS knockdown has a differential effect on VSMC and EC proliferation. MARCKS knockdown results in a p27^kip1^-dependent arrest of VSMC proliferation. In these cells p27^kip1^ is trapped in the nucleus resulting in both increased total p27^kip1^ expression and increased relative nuclear protein expression. Likely both effects are important in effecting the cell cycle arrest. This is the first report that MARCKS exerts a differential effect on proliferation through regulation of KIS. Finally, and most significantly, our *in vitro* observations of the effects of MARCKS knockdown were also observed *in vivo*. siRNA-mediated MARCKS knockdown resulted in a significantly less robust VSMC proliferative response to vascular injury while simultaneously decreasing the time to restoration of the barrier function of the endothelium. This differential response makes MARCKS is an ideal potential target for the prevention of vascular proliferative diseases.

## Supporting Information

S1 Dataset The data presented in this manuscript are found in the S1 Dataset. The data for each figure is presented as an individual spreadsheet in the workbook.
(XLSX)

## Abbreviations

BrdU: bromdeoxyuridine
CAEC: coronary artery endothelial cell
CASMC: coronary artery smooth muscle cell
CDKIs: cyclin-dependent kinase inhibitors
DAPI: 4’,6-diamidino-2-phenylindole
DMEM: Dulbecco’s modified Eagle medium
EC: endothelial cell
FACS: fluorescence-activated cell sorting
FBS: fetal bovine serum
GAPDH: glyceraldehyde 3-phosphate dehydrogenase
KIS: kinase interacting with stathmin
MARCKS: myristolated alanine-rich C kinase substrate
PBS: phosphate buffered saline
PKC: protein kinase C
PI: propridium iodide
RT-PCR: reverse transcription polymerase chain reaction
VSMC: vascular smooth muscle cell
